# A Multiprotein Complex Anchors Adhesive Holdfast at the Outer Membrane of Caulobacter crescentus

**DOI:** 10.1128/JB.00112-19

**Published:** 2019-08-22

**Authors:** Nina I. Sulkowski, Gail G. Hardy, Yves V. Brun, Tanmay A. M. Bharat

**Affiliations:** a Sir William Dunn School of Pathology, University of Oxford, Oxford, United Kingdom; b Central Oxford Structural and Molecular Imaging Centre, Oxford, United Kingdom; c Department of Biology, Indiana University, Bloomington, Indiana, USA; d Département de Microbiologie, Infectiologie et Immunologie, Université de Montréal, Montréal, Québec, Canada; Geisel School of Medicine at Dartmouth

**Keywords:** *Caulobacter*, adhesion, cryo-EM, cryo-ET, electron cryotomography, holdfast, polysaccharides, subtomogram averaging

## Abstract

Adhesion allows microbes to colonize surfaces and is the first stage in biofilm formation. Stable attachment of the freshwater alphaproteobacterium Caulobacter crescentus to surfaces requires an adhesive polysaccharide called holdfast, which is synthesized at a specific cell pole and ultimately found at the tip of cylindrical extensions of the cell envelope called stalks. Secretion and anchoring of holdfast to the cell surface are governed by proteins HfsDAB and HfaABD, respectively. The arrangement and organization of these proteins with respect to each other and the cell envelope, and the mechanism by which the holdfast is anchored on cells, are unknown. In this study, we have imaged a series of C. crescentus mutants using electron cryotomography, revealing the architecture and arrangement of the molecular machinery involved in holdfast anchoring in cells. We found that the holdfast is anchored to cells by a defined complex made up of the HfaABD proteins and that the HfsDAB secretion proteins are essential for proper assembly and localization of the HfaABD anchor. Subtomogram averaging of cell stalk tips showed that the HfaABD complex spans the outer membrane. The anchor protein HfaB is the major component of the anchor complex located on the periplasmic side of the outer membrane, while HfaA and HfaD are located on the cell surface. HfaB is the critical component of the complex, without which no HfaABD complex was observed in cells. These results allow us to propose a working model of holdfast anchoring, laying the groundwork for further structural and cell biological investigations.

**IMPORTANCE** Adhesion and biofilm formation are fundamental processes that accompany bacterial colonization of surfaces, which are of critical importance in many infections. Caulobacter crescentus biofilm formation proceeds via irreversible adhesion mediated by a polar polysaccharide called holdfast. Mechanistic and structural details of how the holdfast is secreted and anchored on cells are still lacking. Here, we have assigned the location and described the arrangement of the holdfast anchor complex. This work increases our knowledge of the relatively underexplored field of polysaccharide-mediated adhesion by identifying structural elements that anchor polysaccharides to the cell envelope, which is important in a variety of bacterial species.

## Abstract (precis)

Adhesion and biofilm formation are fundamental processes that accompany bacterial colonization of surfaces, which are of critical importance in many infections. Caulobacter crescentus biofilm formation proceeds via irreversible adhesion mediated by a polar polysaccharide called holdfast. Mechanistic and structural details of how the holdfast is secreted and anchored on cells are still lacking. Here, we have assigned the location and described the arrangement of the holdfast anchor complex. This work increases our knowledge of the relatively underexplored field of polysaccharide-mediated adhesion by identifying structural elements that anchor polysaccharides to the cell envelope, which is important in a variety of bacterial species.

## INTRODUCTION

Adhesion is essential for bacteria to associate with both abiotic and biotic surfaces and is required for biofilm formation. Bacterial adhesion is facilitated by proteinaceous appendages such as pili and fimbriae, as well as by extracellular polysaccharide adhesins (1). Both Gram-positive and Gram-negative bacteria produce polysaccharide adhesins (2, 3), such as the polysaccharide intercellular adhesin (PIA) and poly-β-1,6-*N*-acetylglucosamine adhesin (PGA). These secreted polysaccharides have a profound impact on adhesion and biofilm formation (4). While the importance of exopolysaccharides in bacteria has been recognized (5), the mechanism by which exopolysaccharide adhesins (including holdfast) are anchored to cells remains incompletely characterized.

Because Gram-negative bacteria have an outer membrane, biosynthesis, secretion, and anchoring of exopolysaccharides require specialized molecular machinery. Numerous studies have been conducted to elucidate the pathways and enzymes associated with exopolysaccharide biosynthesis in Gram-negative bacteria, but the mechanism of polysaccharide anchoring is only beginning to be clarified. Anchoring of exopolysaccharide capsules has been shown to occur through several mechanisms. Capsules are secreted either via an ABC transporter mechanism or by a Wzx/Wzy translocation complex (6, 7). ABC-translocated capsules associate with the outer membrane in one of two ways. The Escherichia coli K1 capsule, composed of polysialic acid (PSA), is anchored to the outer membrane via a conserved phosphatidylglycerol–poly-3-deoxy-d-*manno*-oct-2-ulsonic acid lipid moiety associated with the reducing end of the polysaccharide (8). A second group of bacteria with ABC-translocated capsules, including the Salmonella enterica serovar Typhi Vi antigen, utilize a modification of the lipid A pathway to anchor the polysaccharide (9). The E. coli group I K30 capsule is one of the best-studied examples of an exopolysaccharide secreted via the Wzy-dependent pathway. Wzi is an outer membrane protein that is believed to function as a lectin and bind K30 capsule on the cell surface (10). While many bacteria have Wzi-like proteins associated with their *wzy*-dependent polysaccharide loci, some bacteria do not, which suggests that there still remain other uncharacterized mechanisms of polysaccharide anchoring. The best-studied and described anchoring systems thus far are related to capsular polysaccharides; however, there is a growing list of bacterial species that have polarly expressed polysaccharide adhesins (11–14). The mechanisms of anchoring in these polar polysaccharides are poorly understood and largely underexplored.

Stable attachment of Caulobacter crescentus to surfaces requires an adhesive polysaccharide known as holdfast, localized at the tip of a polar cytoplasmic extension of the cell envelope known as the stalk (15). The holdfast possesses the highest tensile strength of any known biological or synthetic adhesive and demonstrates remarkable versatility in substrate binding (16). The holdfast is a complex, multilayered structure comprised of a mixture of molecules including *N*-acetyl-d-glucosamine (NAG) polysaccharides and proteins and DNA whose identity remains unknown (16). Studies using atomic force microscopy nanoindentation have shown that the holdfast possesses a stiff core surrounded by a flexible polymeric brush layer (16). The outer brush layer is sensitive to proteinase K and DNase I treatment; however, this treatment is insufficient to abolish adhesion or achieve complete removal of the brush layer (16). Therefore, the precise composition and structure of the holdfast remain unknown.

**FIG 1:**
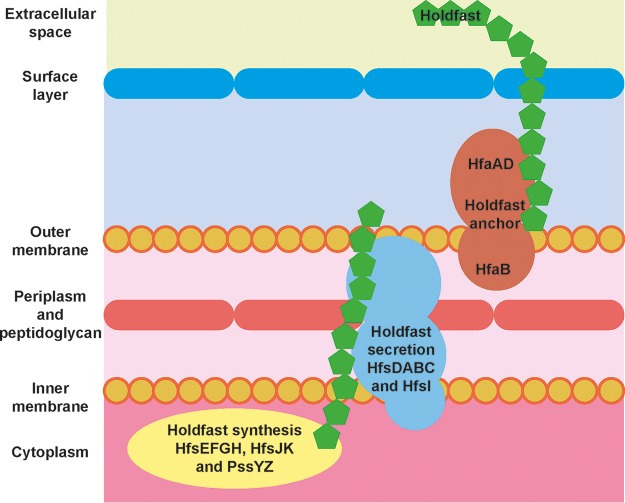
Schematic model of the C. crescentus holdfast synthesis, secretion, and anchor machinery. The holdfast synthesis and polymerization machinery is comprised of proteins (PssY, PssZ, HfsEFGH, HfsJ, and HfsK) from several unlinked loci, all located in the cytoplasm or at the inner membrane of the cell. These proteins are responsible for manufacturing holdfast polysaccharide (green). The holdfast polymerases (HfsC and HfsI) and the holdfast secretion complex (HfsDAB) shuttle holdfast from the inner membrane and across to the outer membrane of the cell envelope. Holdfast polysaccharide is anchored on the cell surface by the holdfast anchor proteins (HfaABD). HfaB is located on the periplasmic side of the outer membrane, while HfaAD proteins are present on the outside.

The holdfast biosynthesis and secretion machinery are encoded by two major loci, *hfsEFGH* and *hfsDABC*, and several unlinked genes (17–19). The *hfsEFGH* locus (Fig. 1), as well as the unlinked genes *pssY*, *pssZ*, *hfsJ*, and *hfsK*, encode a set of cytoplasmic proteins responsible for the synthesis and modification of the exopolysaccharide (19). The Wzy polysaccharide polymerase is encoded by *hfsC* and a paralog, *hfsI*. The *hfsDAB* genes encode a secretion complex believed to span the inner and outer membrane of the C. crescentus cell envelope (Fig. 1), which facilitates the translocation of holdfast from the cytoplasm to the bacterial cell surface (17).

Holdfast is anchored at the C. crescentus stalk tip via the holdfast anchor complex, encoded by the genes *hfaA*, *hfaB*, and *hfaD* (18, 20–22). In line with an anchor function, HfaA, HfaB, and HfaD are enriched in the outer membrane of the cell and localized to the tip of stalks (20, 22). Further, deletion of any of the anchor proteins results in holdfast shedding and decreased adherence and biofilm formation (20–22). HfaA shares similarity to the curlin monomer CsgA and forms SDS- and heat-resistant multimers—consistent with the notion that they are amyloid-like proteins (20). HfaA multimer formation is dependent on the presence of HfaD, which shares limited sequence similarity to surface layer proteins and other adhesins (23). HfaB shares similarity with the CsgG translocon, responsible for the secretion of CsgA across the outer membrane (24). Although the precise function of each anchor component is not known, targeting of HfaA and HfaD to the outer membrane is reliant on HfaB, suggesting that HfaB may form an outer membrane pore for their secretion (20).

To study the arrangement and organization of the holdfast anchor complex, we have used electron cryotomography (cryo-ET) and subtomogram averaging, which support high-resolution structural and cell biology investigations inside bacterial cells (25, 26). By imaging a series of holdfast synthesis, secretion, and anchor complex mutants, we provide insights into the molecular machinery involved in holdfast anchoring on C. crescentus cells, establishing the basis for future structural and cell biology studies on bacterial polysaccharide-mediated adhesion.

## RESULTS

### The holdfast anchor complex is associated with the outer membrane.

**FIG 2:**
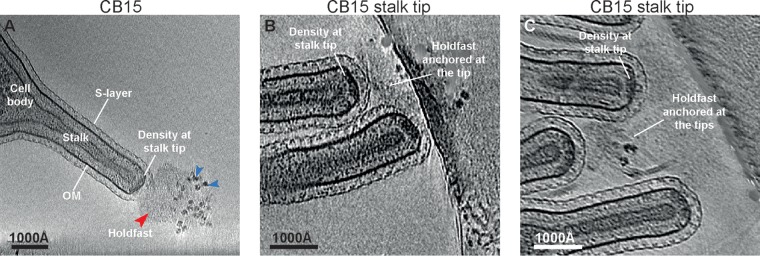
Identification of the cryo-EM density corresponding to the holdfast anchor complex. (A) A virtual slice through a tomogram of a Caulobacter crescentus CB15 cell. The holdfast is visible near the stalk tip (red arrowhead), where it appears anchored to a density directly underneath the outer membrane (marked). The holdfast displays visible granularity (blue arrowheads). (B and C) Zoomed view of the CB15 stalk tips showing the presence of the holdfast anchor complex together with the holdfast.

To understand the architecture and arrangement of the holdfast on the surface of cells and to study how it is anchored at the cell envelope, we imaged a series of C. crescentus CB15 mutants using cryo-ET. First, wild-type CB15 C. crescentus cells were grown to mid-log phase in peptone-yeast extract (PYE) medium and imaged using cryo-ET (see Materials and Methods). It is established that holdfast polysaccharide is located at the tips of the stalks of C. crescentus cells (15). In line with this, inspection of cryotomograms of CB15 stalks revealed the presence of a diffuse density at the tip of the stalk (Fig. 2A, red arrowhead), corresponding to the holdfast, which protruded out of the S-layer of C. crescentus cells into the extracellular milieu. To improve the frequency of imaging stalk tips by cryo-ET, we utilized a stalk shedding mutant (CB15 NY111d1 or Abs2 here; see Table S1 in the supplemental material), which lacked a periplasmic phosphate-binding protein required for phosphate uptake, to form long stalks in rich medium (27, 28). Stalks could then be purified away from the larger, thicker cell bodies, increasing the number of stalk tips available for imaging. In many tomograms, multiple stalks were attached to each other via their holdfast density, forming rosettes (Fig. 2B and C), characteristic of the *Caulobacter* species (29). The holdfast appears to be a complex, multilayered structure with visible granularity (Fig. 2A, blue arrowheads), in agreement with previous data (16).

The holdfast density seemed to emanate from a thick, dense region situated on the periplasmic side of the outer membrane of the stalk tip (Fig. 2A to C; see also Movie S1). In single cryo-ET slices, this cryo-electron microscopy (cryo-EM) density spanned a large percentage of the width of the stalk tip, coating the hemispherical stalk tip uniformly. We found that the holdfast was attached to the stalk tip of all cells possessing the cryo-EM density beneath the outer membrane (Table 1). Therefore, the dense region is likely the site of the complex that anchors the holdfast to the cell surface (20).

**TABLE 1:** Occurrence of anchor and holdfast density in tomograms^*a*^

Strain	% density (*n*)	
	Anchor at stalk tip	Holdfast polysaccharide
CB15 (YB2811)	94 (18)	94 (18)
*hfsG* strain (YB7793)	96 (27)	0 (27)
*hfsDAB* strain (YB7795)	0 (19)	0 (19)
*hfaB* strain (YB7797)	6 (51)	0 (51)
*hfaA* strain (YB8679)	100 (6)	17 (6)
*hfaD* strain (YB8680)	84 (6)	0 (6)
*hfaAD* strain (YB8681)	93 (15)	20 (15)

a All stalk tips in all electron cryotomograms of all strains were visually inspected for the presence of the density of anchor complexes and the holdfast polysaccharide. The percentage of tips with the densities present is reported in the table.

### Determinants of holdfast anchor complex assembly at the outer membrane.

Holdfast biosynthesis and secretion are encoded by two major loci, *hfsEFGH* and *hfsDAB* (23), while the *hfaABD* genes are required for anchoring the holdfast to the cell envelope (20) (Fig. 1). To determine if the observed cryo-EM density beneath the outer membrane was attributable to the anchor complex rather than the secretion complex or the base of the holdfast (i.e., to assign density to molecules), we regenerated deletions in genes contributing to holdfast production in the C. crescentus CB15 Abs2 background (Table S1) and observed the effect of the mutations on cellular ultrastructure using cryo-ET. This included strains with deletions of holdfast synthesis and secretion genes (*hfsG* and *hfsDAB*), as well as the holdfast anchor (*hfaA*, *hfaB*, and *hfaD*) genes.

**FIG 3:**
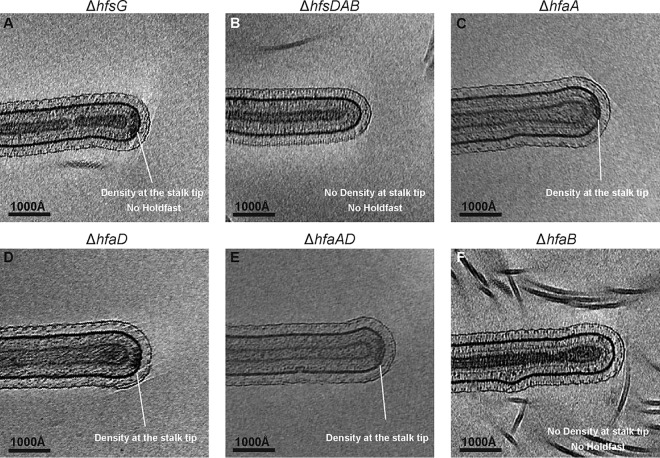
The anchor complex assembly at the outer membrane depends on the holdfast secretion proteins (HfsDAB) and is primarily comprised of HfaB. (A) The cryo-EM density underneath the outer membrane is observed in cells deficient in holdfast synthesis (Δ*hfsG*), indicating that it is not made of polysaccharide but rather a molecular complex associated with the holdfast. (B) Deletion of the holdfast secretion genes, *hfsDAB*, leads to a complete loss of both the density underneath the outer membrane and the holdfast polysaccharide. (C to E) The anchor complex density is observed in stalk tips of the Δ*hfaA*, Δ*hfaD*, and Δ*hfaAD* deletion mutants. (F) A strain with deletion of the holdfast anchor gene, *hfaB*, shows loss of the density underneath the outer membrane at the stalk tip.

First, to determine if the cryo-EM density at the stalk tip is present in the absence of the holdfast, an *hfsG* deletion strain with a deletion in a component of the holdfast synthesis pathway was imaged. Cells lacking HfsG, a putative cytoplasmic glycosyltransferase, are unable to synthesize the holdfast polysaccharide (19). Previous studies have shown that the holdfast anchor is retained at the stalk tip of an Δ*hfsG* deletion strain (30). In line with this, deletion of *hfsG* led to a loss of the holdfast at the stalk tip, while the cryo-EM density underneath the outer membrane was retained (Fig. 3A and Table 1; see also Movie S2). This confirmed that the periplasmic cryo-EM density is distinct from the holdfast itself and is most likely part of the holdfast anchor complex. Next, a mutant lacking the entire *hfsDAB* operon was imaged. The HfsDAB protein complex is predicted to span the inner and outer membrane of C. crescentus and is responsible for the translocation of holdfast polysaccharide to the cell surface (17). Both the holdfast and the cryo-EM density underneath the outer membrane were lost in the Δ*hfsDAB* mutant (Fig. 3B; Table 1). These results confirm the previous observation that the HfsDAB proteins are important for the correct localization of the holdfast anchor complex (20, 30) and suggest that HfsDAB may partly contribute to the cryo-EM density underneath the outer membrane.

To explore which of the holdfast anchor proteins (HfaABD) contribute to the cryo-EM density underneath the outer membrane, we next turned to a panel of *hfaABD* deletion mutants. These *hfa* mutants are able to synthesize holdfast normally; however, they have reduced ability to retain it at the cell surface, causing holdfast shedding into the medium (20). As expected, the holdfast was poorly retained at the stalk tips in all *hfa* deletion—Δ*hfaA*, Δ*hfaD*, Δ*hfaAD*, and Δ*hfaB*—strains, confirming that these anchor complex proteins are required for holdfast anchoring (Fig. 3C to F, Table 1, and Fig. S1A to H). Furthermore, cryo-ET revealed that the density was retained beneath the outer membrane of Δ*hfaA* and Δ*hfaD* single anchor mutants and the Δ*hfaAD* double anchor mutant (Fig. 3C to E, Table 1, and Fig. S1A to F). The cryo-EM density beneath the outer membrane in these strains possessed the same microscopic appearance as that seen in the wild-type CB15 stalk tips (Fig. 2). To investigate if deletion of *hfaA*, *hfaD*, or *hfaAD* resulted in subtle changes in localization, we measured the percent coverage of the cryo-EM density along the width of the stalk tip in wild-type CB15 and Δ*hfaA*, Δ*hfaD*, and Δ*hfaAD* strains. We found no significant difference between strains (overall average, 57.6%; *P* value of differences, 0.99), reaffirming these proteins are not the major component of the cryo-EM density on the periplasmic side of the outer membrane. In contrast, the density beneath the outer membrane was completely lost in the Δ*hfaB* mutant (Fig. 3F, Table 1, Fig. S1G to H, and Movie S3). Collectively, these findings suggest that the holdfast anchor complex density on the periplasmic side of the outer membrane is comprised primarily of the holdfast anchor protein HfaB, or HfaB in complex with the holdfast secretion machinery, HfsDAB. Previous studies suggest that HfaB may play a role in holdfast biogenesis possibly through the stabilization of the secretion complex (30). However, HfsD has been shown to localize to the stalk tip in the absence of HfaB (30), suggesting that the cryo-EM density is primarily due to the presence of HfaB. It is therefore unclear why the secretion complex is not visible in the Δ*hfaB* mutant.

### The holdfast anchor complex spans the outer membrane.

**FIG 4:**
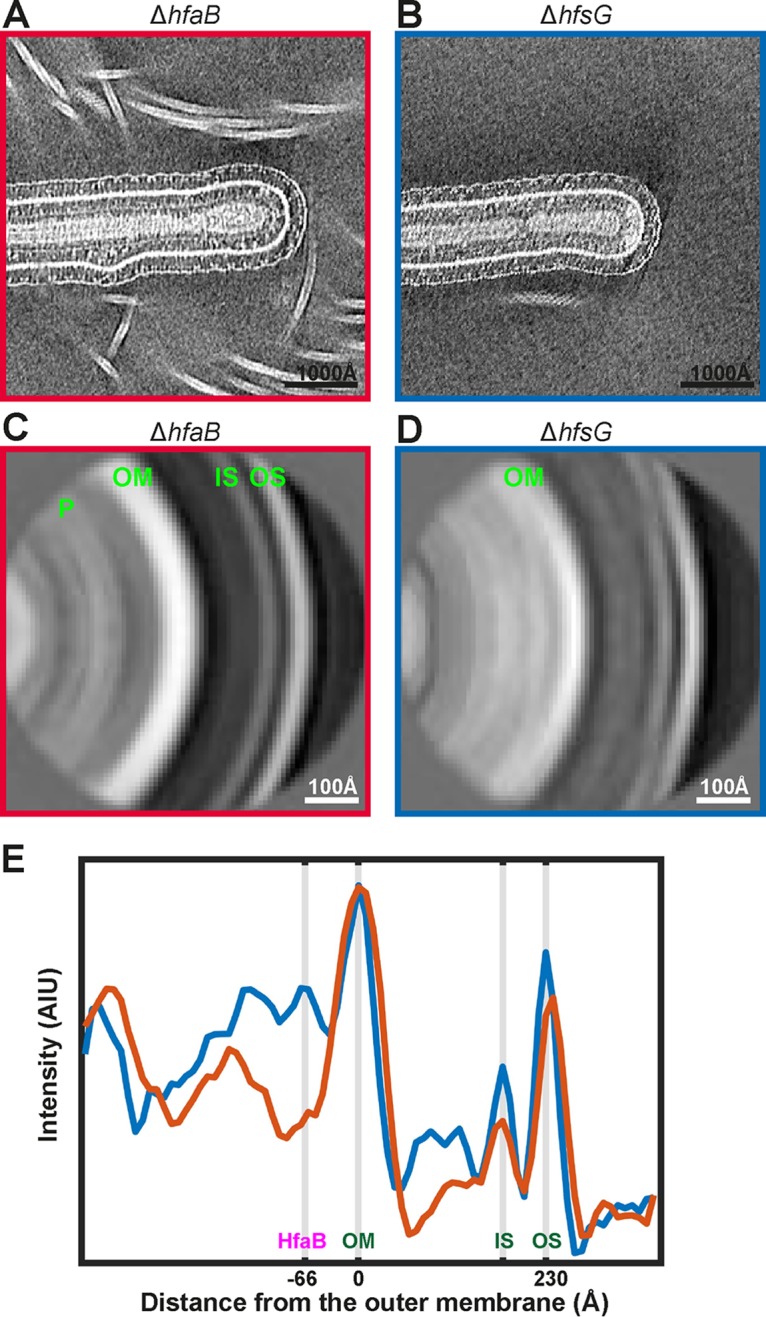
Comparison of stalk tips with and without the holdfast anchor complex. (A) Stalk tip of the Δ*hfaB* strain that lacks the anchor complex. (B) Stalk tip of the Δ*hfsG* strain that possesses a visible anchor complex. (C and D) Subtomogram averaging analysis of tips without and with the anchor complex (see Materials and Methods). The density layers corresponding to the outer S-layer (OS), inner S-layer (IS), outer membrane (OM), and the peptidoglycan (P) have been marked. (E) Plots of the pixel intensity across the subtomogram averages, for the Δ*hfaB* (red curve) and the Δ*hfsG* (blue curve) strains. The outer S-layer, inner S-layer, and outer membrane have been marked as in panel C. The density underneath the outer membrane corresponding to HfaB has also been marked.

In order to understand the architecture of the holdfast anchor complex, cryo-electron tomograms of anchor-complex-lacking (Δ*hfaB*) and anchor-complex-containing (Δ*hfsG*) strains (Fig. 4A and B) were analyzed using subtomogram averaging analysis (see Materials and Methods). Regions of tomograms containing the anchor complex density were selected manually. Periodic and overlapping subtomograms along the outer membrane of the cell stalk were extracted (26, 31). The extracted subtomograms were rotated to vertical orientation using the angle between successive overlapping subtomograms, in order to improve the accuracy of alignments in the analysis. Extracted subtomograms were collapsed onto a two-dimensional (2D) image and then aligned and averaged using a regularized likelihood algorithm implemented in the RELION software (32, 33). The accuracy of alignment was judged by carefully inspecting the class averages as well as by inspecting the Euler angles assigned to individual subtomograms by the algorithm.

Class average images showed distinct peaks in the density corresponding to the outer S-layer lattice, inner S-layer domains, the outer membrane, and the peptidoglycan layer in both the Δ*hfaB* and Δ*hfsG* mutants (Fig. 4C and D). The distance between the outer S-layer lattice and outer membrane was 230 Å, in agreement with previous tomographic studies of the C. crescentus cell envelope (34). Thus, the distance between the S-layer and outer membrane of the cell envelope was unaffected in the presence or absence of the holdfast anchor complex. Further inspection of subtomogram class averages revealed a strong density lining the periplasmic side of the outer membrane in the Δ*hfsG* mutant (Fig. 4E, blue curve), corresponding to the periplasmic part of the holdfast anchor complex. As expected from visual inspection of the raw data, these densities were absent underneath the outer membrane of Δ*hfaB* mutant (Fig. 4E, red curve).

As a further control, we performed subtomogram averaging on Δ*hfsDAB* stalks that also lack the cryo-EM density underneath the outer membrane. We wanted to test whether this strain phenocopies the Δ*hfaB* mutant. Inspections of tomograms revealed that Δ*hfsDAB* stalks, in contrast with Δ*hfaB* (Fig. S2A), displayed slightly deformed stalk tip morphologies, forming narrower, tapered, and cone-shaped stalk tips in many instances (Fig. S2B to D). The consequence of aberrant stalk morphology is evident in the final class average where the outer membrane curvature in the Δ*hfsDAB* mutant is greater (Fig. S2F) than that of the Δ*hfaB* mutant (Fig. S2E). The variation in membrane curvature limits application and comparison of the Δ*hfsDAB* mutant with Δ*hfaB* using subtomogram averaging. Change in stalk morphology upon Δ*hfsDAB* deletion may suggest that the secretion complex plays a role in stabilizing structures at the outer membrane of the stalk tip.

**FIG 5:**
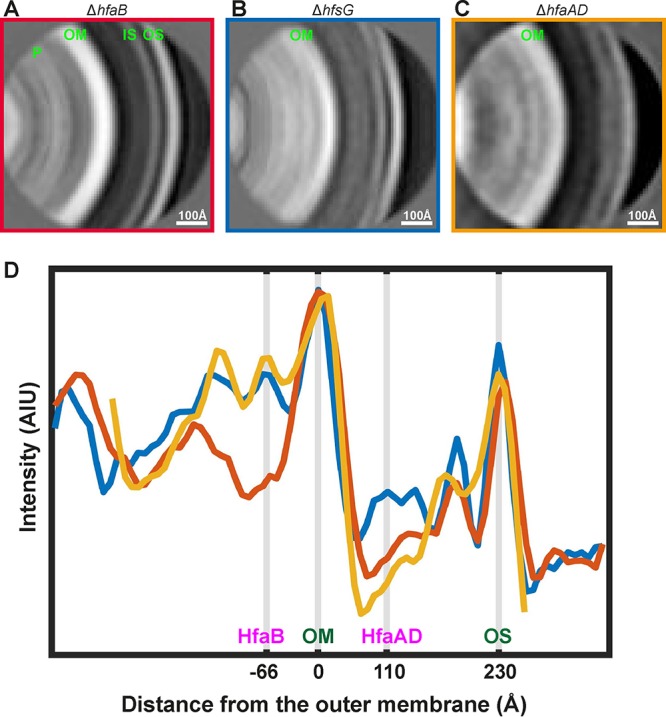
The holdfast anchor complex spans the outer membrane of C. crescentus. (A to C) Subtomogram averaging analysis of stalk tips from the Δ*hfaB*, Δ*hfsG*, and Δ*hfaAD* strains. Panels A and B are the same as in Fig. 4, shown here for comparison with panels C and D (see Materials and Methods). Density layers corresponding to the outer S-layer (OS), inner S-layer (IS), outer membrane (OM), and the peptidoglycan (P) have been marked in panel A. (D) Plots of the pixel intensity across the subtomogram averages for the Δ*hfaB* (red curve), the Δ*hfsG* (blue curve), and the Δ*hfaAD* (orange curve) strains. The outer S-layer, the outer membrane, and the locations of the HfaABD proteins have been marked.

On comparing the linear density profiles of the two class averages presented in Fig. 4E, an additional cryo-EM density peak was observed on the extracellular side of the outer membrane in the Δ*hfsG* mutant, which was absent in the Δ*hfaB* mutant. To test whether this density could be attributable to HfaAD, we repeated the same subtomogram analysis for the Δ*hfaAD* double mutant (Fig. 5C). The peak on the extracellular side of the outer membrane was not observed in this mutant (Fig. 5D, orange curve), while the HfaB peak on the periplasmic side was retained. This suggests that the broad peak of density on the extracellular side of the outer membrane is made up of HfaAD proteins either alone or in conjunction with the base of the holdfast.

The center of the HfaB density is situated ∼66 Å away from the center of the outer membrane (Fig. 5D), which is comparable to the published size of CsgG (35). HfaB appears as a sharp peak (Fig. 5D, blue curve), and it is clearly discernible by visual inspection of tomograms. The center of the extracellular density corresponding to HfaAD proteins is ∼110 Å away from the outer membrane and forms a broad peak (Fig. 5D, compare blue and orange curves). This indicates that the anchor proteins likely form extended oligomers at the coating on the outer leaflet of the outer membrane; however, further structural analysis will be needed to confirm this. Taken together, the data show that the holdfast anchor complex spans the outer membrane with different components of the complex situated on both sides of the outer membrane.

## DISCUSSION

Attachment of bacteria to surfaces via proteinaceous adhesins such as pili and fimbriae has been extensively studied; however, comparatively less is known about polysaccharide-mediated adhesion. For the Gram-negative bacterium C. crescentus, initial adhesion of swarmer cells is mediated by pili and flagella (36–38). Stable, irreversible attachment is then achieved by the holdfast (17, 18, 22).

In this study, we have identified the location and described the arrangement of the holdfast anchor complex at the C. crescentus stalk tip. The components of the holdfast anchor complex are localized on both sides of the outer membrane of the stalk. The sharp, regular cryo-EM density of the anchor complex on the inner side of the outer membrane is comprised primarily of the holdfast anchor protein HfaB, consistent with previous genetics and cell biology studies (20). The cryo-EM density corresponding to the anchor complex is lost upon Δ*hfsDAB* deletion. Previous work has suggested that HfaB and HfsD of the holdfast secretion complex interact directly, since proper localization of HfaB requires HfsD (30). In line with this, localization of HfaB at the stalk tip was disrupted by the Δ*hfsDAB* deletion (20, 30). It remains unclear if other components of the HfsDAB complex associate with HfaB, and systematic deletion of individual secretion genes may help to elucidate details of this interaction in the future.

Both HfaA and HfaD are known to localize at the outer membrane of C. crescentus cells (20), and they were found to contribute to the broad cryo-EM density associated with the extracellular side of the outer membrane in this study. Immunofluorescence microscopy has shown that both proteins are localized on the cell surface (20); therefore, they are not expected to contribute to the cryo-EM density on the periplasmic side of the outer membrane. Further structural and cell biology investigations will be needed to understand which components of the HfaABD complex directly tether sugar moieties of holdfast to cells.

HfaB has a predicted structural similarity with CsgG, an outer membrane porin responsible for the translocation of the curli amyloid subunits to the cell surface (24). As HfaB is closely associated with the outer membrane of the C. crescentus stalk (with the center of the HfaB peak only ∼66 Å away) and is thought to interact with the holdfast secretion complex, HfsDAB, it is highly likely that a part of HfaB is buried in the outer membrane and plays a similar role in secretion to CsgG. Indeed, the localization of HfaA and HfaD at the cell surface depends on the presence of HfaB supporting its function as a putative translocon (20).

The mechanism by which polysaccharide adhesins are anchored to the cell body in C. crescentus and many other bacterial species remains largely uncharacterized. Since polysaccharides play a crucial role in adhesion and biofilm formation of many bacteria, there is an urgent need to uncover fundamental principles of polysaccharide anchoring, adhesion, and their regulatory effect in bacteria. This study thus sets the framework for future structural and cell biology studies of the holdfast anchor complex and reveals insights into a fundamental but underexplored area, crucial in improving understanding of polysaccharide-mediated adhesion in bacteria.

## MATERIALS AND METHODS

### Bacterial strains and growth conditions.

Bacterial strains and plasmids used in this study are listed in Table S1 in the supplemental material. C. crescentus strains were grown in peptone-yeast extract (PYE) medium (11) at 30°C, with antibiotic and carbon supplements at the indicated concentrations when necessary: kanamycin (20 μg/ml [plate] or 5 μg/ml [broth]) and nalidixic acid (20 μg/ml [plate]). Escherichia coli strains were cultured at 37°C in Luria-Bertani (LB) medium. LB medium was supplemented with kanamycin (50 μg/ml or 25 μg/ml [plate]) when necessary.

### DNA manipulations and sequencing.

All primers used in this study are listed in Table S2 and were purchased from Eurofins Genomics (Louisville, KY). PCR products were purified using QIAquick spin columns (Qiagen, Valencia, CA) according to procedures recommended by the manufacturer. Chromosomal DNA was isolated using the Promega Magic MiniPrep DNA purification system (Promega, Madison, WI) using the manufacturer’s instructions. DNA sequencing was performed by Eurofins Genomics. Sequence data were analyzed using Sequencher 5.4.6 software (Gene Codes Corporation, Ann Arbor, MI).

### Construction of single and combination deletion mutants.

All deletions were generated in two steps using homologous recombination using upstream and downstream fragments of a gene cloned into a nonreplicating plasmid, pNPTS138 or pNPTS139, which carries a kanamycin resistance gene cassette (*nptI*), along with the *sacB* cassette, which confers sucrose sensitivity as previously described (39). The deletion mutants were confirmed by colony PCR using the primers used to clone the upstream and downstream fragments and verified by sequencing (Table S2). Plasmids were introduced into C. crescentus by conjugation.

### Transduction of *pstS*::mini-Tn*5*.

Transduction was performed as previously described in reference 40. A phiCR30 phage lysate of CB15N *pstS*::mini-Tn*5* was used to transduce the CB15 Abs2 deletion mutants. Transductants were grown on PYE with kanamycin to select for the *pstS*::mini-Tn*5* mutation.

### Cryo-EM sample preparation.

Caulobacter crescentus strains were grown in PYE medium supplemented with 5 μg/ml kanamycin (except for CB15) at 30°C in a shaking incubator to an optical density (600 nm) of 0.5. Fifteen microliters of liquid culture was mixed with 1 μl of 10-nm gold fiducial beads (CMC Utrecht) and applied to freshly glow-discharged 200-mesh Cu/Rh Quantifoil (3.5/1) grids. Grids were plunge-frozen into liquid ethane using an FEI Vitrobot (Mark IV) and stored in liquid nitrogen until cryo-ET imaging.

### Cryo-EM and cryo-ET data collection.

Initial cryo-EM assessment of samples was conducted on an FEI Talos Arctica 200-kV cryo-transmission electron microscope (cryo-TEM) fitted with a Falcon 3 direct electron detector. Cryo-ET data collection was performed on Titan Krios microscopes running at 300 kV (FEI/ThermoFisher), each fitted with a Quantum energy filter (slit width, 20 eV) and a K2 direct electron detector (Gatan) running in counting mode with a dose rate of ∼8 electrons/pixel/s at the camera level. Tilt series were collected between ±60° in two directions at a 1° increment using SerialEM (41). A total dose of ∼121 e^−^/Å^2^ was applied, and data were sampled at pixel sizes of either 4.4 Å or 4.2 Å (scale bars are shown in each figure panel).

### Image processing of stalk tips.

Following data collection, contrast transfer function (CTF) parameters for each image in the tilt series were estimated using CTFFIND (42). Tilt series alignment was performed using gold fiducial markers in IMOD (43). Gold fiducial densities were erased before CTF compensation within IMOD, followed by tomographic reconstruction using the Simultaneous Iterative Reconstruction Technique (SIRT) algorithm (43). Two-nanometer-thick tomographic slices are shown in Fig. 2 and 3, except in Fig. 2A, where a 3-nm slice is presented. Periodic sites along the outer membrane of the C. crescentus stalk (containing visible anchor complex densities) were manually selected. A spline was fitted through each set of sites (Matlab), and the in-plane rotation from the spline fit was calculated for subsequent analysis as described previously (31). Briefly, subtomograms were extracted, collapsed onto a 2D image (33) along the fitted spline, and subjected to two-dimensional averaging using regularized likelihood optimization in RELION (32). Two-dimensional collapsed subtomograms from multiple C. crescentus stalk tips were averaged together. Averages were then mirrored across the horizontal and added with the mirror image in order to boost the signal-to-noise ratio, to produce the final averages shown in Fig. 4C and D and Fig. 5A to C. Final averages from different biological samples were then placed on the same spectral profile based on their individual power spectra (44), and the intensities in the average image were normalized to the intensity of the outer membrane. Due to the inherent variability between samples and different stalk tips, only the presence or absence of peaks in the line profiles was interpreted, rather than the absolute intensities.

## Supplementary Material

Supplemental file 1

Supplemental file 2

Supplemental file 3

Supplemental file 4
