# Supplementary material for: A Multiprotein Complex Anchors Adhesive Holdfast at the Outer Membrane of Caulobacter crescentus
Source: J Bacteriol. 2019 Aug 22;201(18):e00112-19. doi: 10.1128/JB.00112-19 (PMC6707917; doi:10.1128/JB.00112-19)
Supplement: Supplemental file 1 [file JB.00112-19-s0004.pdf]

Supplementary Material for Sulkowski *et al*

A multi-protein complex anchors adhesive holdfast at the outer membrane of *Caulobacter crescentus*.

Authors

Nina I. Sulkowski,[a,b,*] Gail G. Hardy,[c,*,#] Yves V. Brun,[c,d] Tanmay A.M. Bharat[a,b,#]

Affiliations

[a] Sir William Dunn School of Pathology, University of Oxford, Oxford OX1 3RE, UK

[b] Central Oxford Structural and Molecular Imaging Centre, Oxford OX1 3RE, UK

[c] Department of Biology, Indiana University, Bloomington, Indiana 47405, USA.

[d] Département de microbiologie, infectiologie et immunologie, Université de Montréal, C.P. 6128, succ. Centre-ville, Montréal (Québec) H3C 3J7, Canada

Running Title

Caulobacter crescentus outer membrane holdfast anchor

Correspondence

# Address correspondence to Gail Hardy, gahardy@indiana.edu or Tanmay A.M. Bharat, tanmay.bharat@path.ox.ac.uk

* N.I.S. and G.G.H. contributed equally to this work.

SUPPLEMENTARY FIGURES

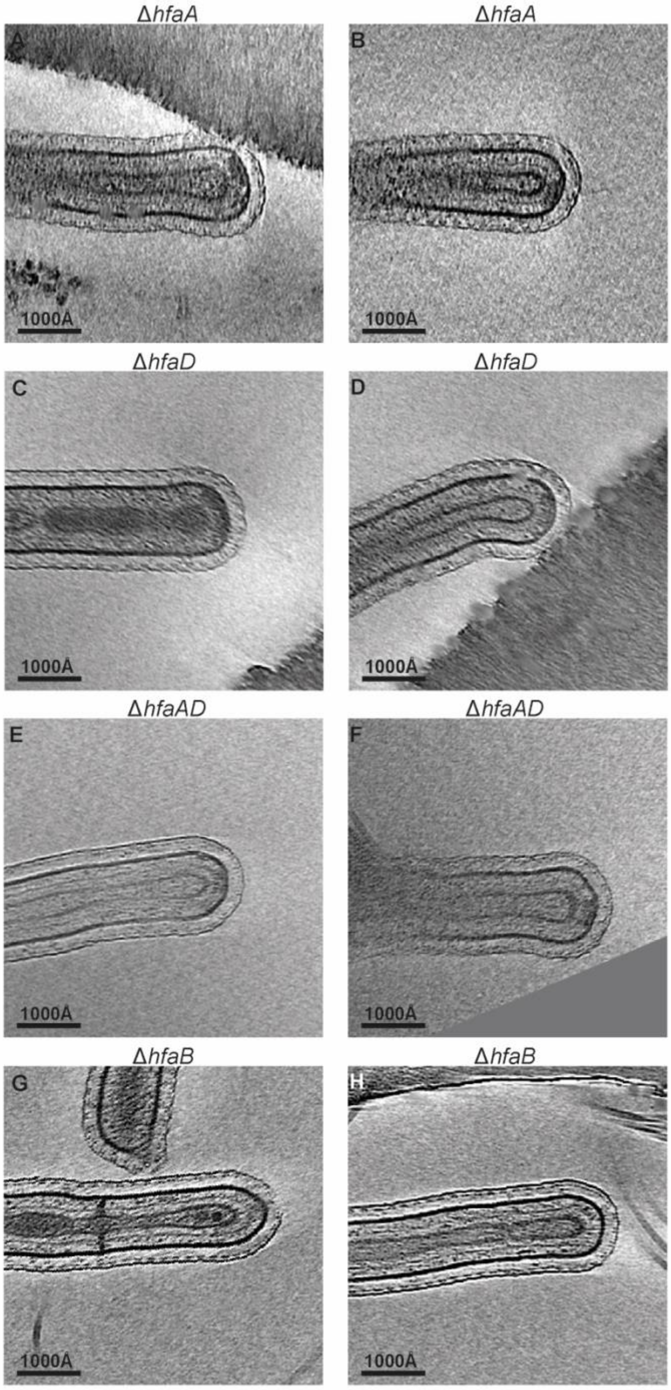

Figure S1. Cryo-ET gallery of *C. crescentus* stalk tips.

Collection of holdfast anchor mutants imaged in this study. (A-B) Δ*hfaA* (C-D) Δ*hfaD* (E-F) Δ*hfaAD* (G-H) Δ*hfaB.*

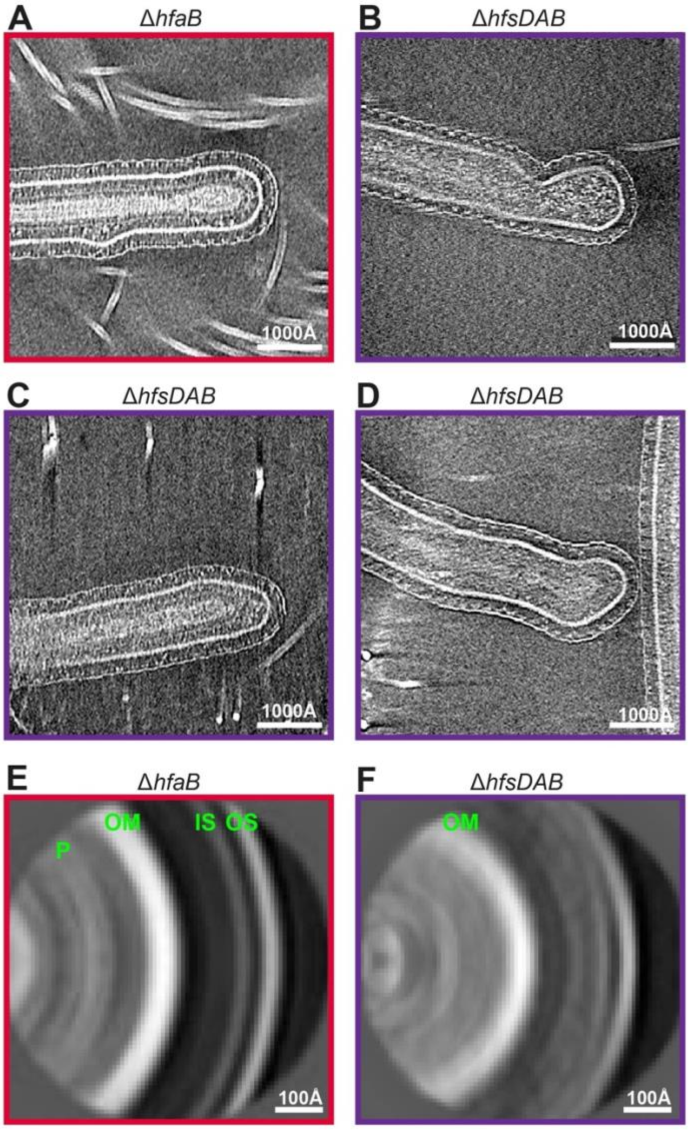

Figure S2. Sub-tomogram averaging of the holdfast secretion complex mutant (Δ*hfsDAB*). (A) Δ*hfaB* strain displays uniform curvature along the stalk tip. Panels A is same as Figure 4A, shown here for comparison with B-D panels. (B-D) Δ*hfsDAB* mutant displays aberrations in stalk morphology, with visible narrowing at various regions near the tip. (E) Sub-tomogram averaging analysis of Δ*hfaB* stalk tips. Panel E is same as in Figure 4C. The density layers corresponding to the outer S-layer (OS), inner S-layer (IS), outer membrane (OM) and the peptidoglycan (P) have been marked. (F) Sub-tomogram averaging analysis of Δ*hfsDAB* stalks reveals increased OM curvature in the final average.

SUPPLEMENTARY TABLES

TABLE S1. Bacterial strains and plasmids used in this study.

Strain or plasmid	Description or construction	Source or reference
**Strains**		
E. coli		
S17-1	*E. coli* 294::RP4-2(Tc::Mu)(KM::Tn*7*)	(1)
Alpha-select™	*deoR endA1 recA1 relA1 gyrA96 hsdR17*(r_k^- m_k^+) *supE44 thi*-1 Δ(*lacZYA-argFV169*) Φ80δ*lac*ZΔ*M15* F^-	Bioline
C. crescentus		
CB15	Wild-type	(2)
YB1109	CB15 NY111d1 is a clone that has the Abs phenotype (stalk abcission Abs2)	(3)
YB2811	CB15 Abs2 *pstS*::miniTn5	(4)
YB767	CB15N *pstS*::miniTn5	(5)
YB7793	CB15 Abs2 *pstS*::miniTn5, Δ*hfsG*	This study
YB7795	CB15 Abs2 *pstS*::miniTn5, Δ*hfsDAB*	This study
YB7797	CB15 Abs2 *pstS*::miniTn5, Δ*hfaB*	This study
YB8679	CB15 Abs2 *pstS*::miniTn5, Δ*hfaA*	This study
YB8680	CB15 Abs2 *pstS*::miniTn5, Δ*hfaD*	This study
YB8681	CB15 Abs2 *pstS*::miniTn5, Δ*hfaA,* Δ*hfaD*	This study
**Plasmids**		
pNPTS138/9	pLitmus 39 derivative; with *nptI, sacB* and RK2 *oriT* sequences*,* and deleted *bla* gene; Km^R	M.R.K. Alley, unpublished
pNPTS138Δ*hfaA*	pNPTS138 parent vector containing 500 bp fragments upstream and downstream of *hfaA*	(6)
pNPTS138Δ*hfaB*	pNPTS138 parent vector containing 500 bp fragments upstream and downstream of *hfaB*	(6)
pNPTS138Δ*hfaD*	pNPTS138 parent vector containing 500 bp fragments upstream and downstream of *hfaD*	(6)
pNPTS138Δ*hfsDAB*	pNPTS138 parent vector containing 500 bp fragments downstream of *hfsD* and downstream of *hfsB*	(6)
pNPTS138Δ*hfsG*	pNPTS138 parent vector containing 500 bp fragments upstream and downstream of *hfsG*	(7)

Table S1. Bacterial strains and plasmids used in this study.

Description of bacterial strains and plasmids used in this study is provided in this table.

TABLE S2. Oligonucleotides used in this study

Oligo Name	Sequence (5' - 3')	Application
FhfaupA	GCGATCACGAAGCTTACGAGGTAG	*hfaA* in-frame deletion
RmhfadownA	GGCGTCAATGAATTCCGAGCGGAT	*hfaA* in-frame deletion
FhfaupD	GGCATCACCAAGCTTAACTACAAC	*hfaD* in-frame deletion
RmHfadownD	GCGGCCTGGGAATTCTAGTCCTGA	*hfaD* in-frame deletion
FupSphhfsG	CTGCCCGCATGCGTCGGCCTC	*hfsG* in-frame deletion
RdwnBamHihfsG	GTTGAGATCGGATCCGTAGGTGAT	*hfsG* in-frame deletion
hfsDHindend	GATCAGCTTAAGCTTCTCCTCAGG	*hfsDAB* in-frame deletion
hfsBHindend	TCCATAGCCAAGCTTAGGCGCCGG	*hfsDAB* in-frame deletion
FhfaBup	GCCTTCACGCCGGGATCCAACAATCTGGGA	*hfaB* in-frame deletion
RmHfadownB	CACGTTGGCGAATTCCGACTGGCT	*hfaB* in-frame deletion

Table S2. Oligonucleotides used in this study

Oligonucleotide sequences used in this study are listed in this table.

SUPPLEMENTARY MOVIE LEGENDS

Movie S1. Tomogram of CB15 cell stalks.

A movie containing sequential z-slices of a reconstructed tomogram of *C. crescentus* CB15 cell stalk tips is presented. This tomogram shows the presence of the holdfast anchor complex at the stalk tip.

Movie S2. Tomogram of a cell stalk tip from the *hfsG* deletion strain.

A movie containing sequential z-slices of a reconstructed tomogram of *C. crescentus* Δ*hfsG* cell stalk tips is presented. This tomogram also shows the presence of the holdfast anchor complex at the stalk tip.

Movie S3. Tomogram of a cell stalk tip from the *hfaB* deletion strain

A movie containing sequential z-slices of a reconstructed tomogram of *C. crescentus* Δ*hfaB* cell stalk tips is presented. This tomogram shows the absence of the holdfast anchor complex at the stalk tip.

SUPPLEMENTARY REFERENCES

1. R. Simon, U. Priefer and A. Puhler. 1983. A Broad Host Range Mobilization System for Invivo Genetic-Engineering - Transposon Mutagenesis in Gram-Negative Bacteria. Bio-Technology 1:784-791.
2. J. S. Poindexter. 1964. Biological Properties and Classification of the Caulobacter Group. Bacteriol Rev 28:231-95.
3. J. S. Poindexter. 1978. Selection for nonbuoyant morphological mutants of Caulobacter crescentus. J Bacteriol 135:1141-5.
4. M. M. Ireland, J. A. Karty, E. M. Quardokus, J. P. Reilly and Y. V. Brun. 2002. Proteomic analysis of the Caulobacter crescentus stalk indicates competence for nutrient uptake. Mol Microbiol 45:1029-41.
5. M. Gonin, E. M. Quardokus, D. O'Donnol, J. Maddock and Y. V. Brun. 2000. Regulation of stalk elongation by phosphate in Caulobacter crescentus. J Bacteriol 182:337-47.
6. G. G. Hardy, R. C. Allen, E. Toh, M. Long, P. J. Brown, J. L. Cole-Tobian and Y. V. Brun. 2010. A localized multimeric anchor attaches the Caulobacter holdfast to the cell pole. Mol Microbiol 76:409-27.
7. E. Toh, H. D. Kurtz and Y. V. Brun. 2008. Characterization of the Caulobacter crescentus Holdfast Polysaccharide Biosynthesis Pathway Reveals Significant Redundancy in the Initiating Glycosyltransferase and Polymerase Steps. J Bacteriol 190:7219-7231.
